# The impact of expert by experience involvement in teaching in a DClinPsych programme; for trainees and experts by experience

**DOI:** 10.1111/hex.13817

**Published:** 2023-07-13

**Authors:** Ellie Kerry, Nicola Collett, Jason Gunn

**Affiliations:** ^1^ Oxford Institute for Clinical Psychology Research University of Oxford Oxford UK; ^2^ Oxford Institute for Clinical Psychology Research University of Oxford Oxford United Kingdom

**Keywords:** Doctorate of Clinical Psychology, experts by experience, involvement, participation, teaching, Trainee Clinical Psychologist

## Abstract

**Introduction:**

There is a growing acknowledgement of the value of creating partnerships between those delivering and those accessing health services. Less is known about this in the context of clinical psychology doctoral training programmes. This study explores the models of involvement of experts by experience (EbEs) in teaching on a DClinPsych course in England; the impact of this both for EbEs and trainee clinical psychologists and whether improvements are required to better meet their needs.

**Methods:**

An audit of current involvement was conducted by reviewing course records. Two survey questionnaires designed around commonly used frameworks of participation and reflective learning were completed by EbEs and trainees. Thematic Analysis was used to evaluate the written feedback from the surveys.

**Results:**

Records of current EbE involvement were found to be lacking in detail and sometimes missing. Key themes extrapolated from the surveys highlighted the importance of EbE involvement in supporting the wellbeing of EbEs and the learning experiences of trainees.

**Conclusions:**

Recommendations with regard to the processes for future involvement of EbEs in teaching are put forward.

**Patient or Public Contribution:**

A carer of a service user was consulted about the design of the participant information sheet, consent form and the survey questionnaire which was sent to the EbEs. A trainee clinical psychologist was also consulted to provide a trainee perspective on the above forms and the survey questionnaire that was sent to trainees. Further to this, the first author's supervisor identifies as a user of physical and mental health services and provided continued supervision and support regarding the direction of the study including the research questions, design, methodology and interpretation of results.

## 1 INTRODUCTION

Experts by experience (EbEs) are people with lived experience of using or caring for someone who uses health or social care services.^1^ EbEs are thought to bring invaluable insights and perspectives, via their personal expertise, to the training of health practitioners.^2^ The importance of encouraging patient participation and co‐production has been emphasised in statutory guidance,^3^ and there is growing recognition of the value of EbE involvement in NHS training programmes such as the Doctorate in Clinical Psychology (DClinPsych).

### 1.1 Impact of involvement for EbEs

**Figure 1:**
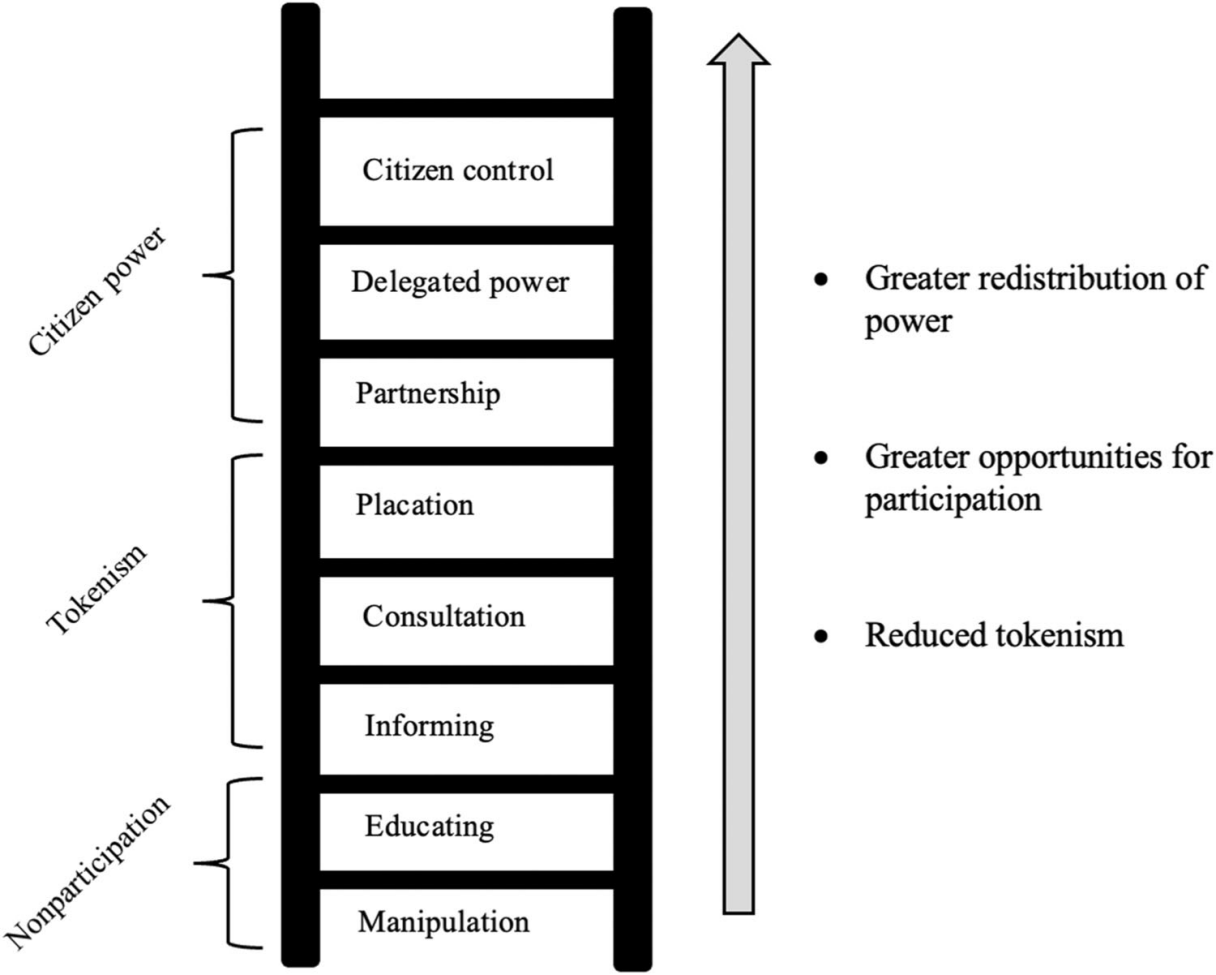
Adaptation of the ‘Ladder of participation’.^8^

Closer partnerships between EbEs and organisations involved in their care can bring about a sense of empowerment, confidence and wellbeing for EbEs.^4^, ^5^ This is thought to in part be due to a breaking down of stigmatising power differentials through greater contact between EbEs and professionals, in the context of equal status.^6^, ^7^ The redistribution of power hierarchies is central to the Ladder of Participation^8^; an eight‐step model that provides a benchmark for understanding different levels of involvement, ranging from ‘manipulation’ to ‘citizen control’ (Figure 1).^8^ It measures the extent to which EbEs are provided with opportunities to exert influence and power in the health system and their own care,^9^ taking account of their personal ‘choice’ over the position they wish to hold.^10^ Despite national guidelines, research studies show that there can be a gap between what is recommended and what is delivered with regard to EbE involvement in healthcare training settings.^11^, ^12^ Therefore, there is an ongoing need to evaluate the level of EbE involvement in clinical psychology training, to ensure that it remains beneficial and meaningful, rather than tokenistic, for those involved.^13^

### 1.2 The impact of involvement for students

**Figure 2:**
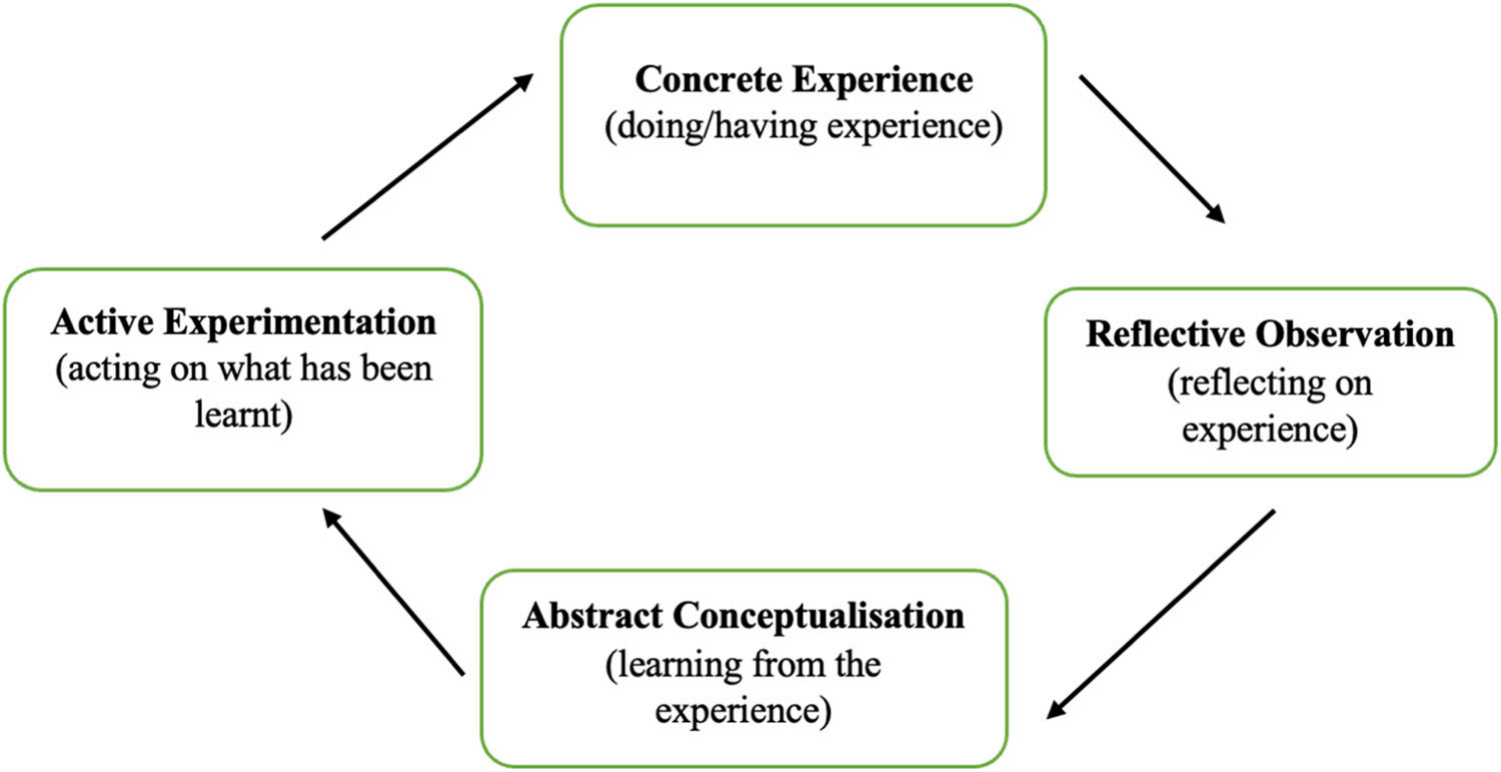
Adaptation of Kolb's reflective learning cycle.^17^

Studies show that students, including clinical psychology doctorate trainees, feel that EbE involvement in their education, can improve their clinical practice by helping them reflect on their therapeutic relationships.^14^, ^15^ The process of learning is promoted by experiences of reflection that occur within a social context.^16^, ^17^ In line with Kolb's reflective cycle (Figure 2), EbE involvement in teaching may offer greater opportunities for learning by reaching students on an emotional level and supporting the transformation of experience into learning and new behaviour.^17^, ^18^ Kolb's reflective cycle has been used to conceptualise the process by which EbE involvement in social work training can promote trainee learning by building greater reflective awareness that can then be taken into their practice.^19^ As yet, this model has not been used to evaluate the impact of EbE involvement in the training of trainee clinical psychologists.

In line with ongoing plans to increase EbE involvement in a UK DClinPsych course and move towards a position of co‐production (Appendix A), this study aimed to explore the following research questions:

1. How are EbEs currently involved in teaching on a DClinPsych course?
2. How are EbEs and trainees impacted by EbE involvement in teaching?
3. What do EbEs and trainees think could be improved with regard to EbE involvement in teaching?

## 2 METHODS

### 2.1 Design

The study employed a qualitative survey design that was approved as a service improvement project by the local NHS Trust. It was guided by the Model for improvement and aimed to gather the information required to generate change ideas and recommendations for EbE involvement in teaching on a DClinPsych course.^20^ The survey approach was chosen to allow for a broader range of perspectives to be collected, compared to an interview approach which may not reach as many participants.

### 2.2 Participants

Two participant groups were recruited by email via a DClinPsych course in the United Kingdom; EbEs and first‐year trainee clinical psychologists. The trainees were all in the first year of a 3‐year Doctorate in Clinical Psychology (DClinPsych) which is a course that incorporates academic teaching, research and clinical placements for trainee clinical psychologists.

The EbEs were recruited from a ‘People's Experience Group' (PEG) that was affiliated with the specific DclinPsych course under assessment in this study. This group included representatives with personal experience accessing services or caring for people accessing services that clinical psychologists work in. The role of the EbEs in this group was to provide lived experience perspectives across a range of areas of the course, including teaching.

### 2.3 Procedure

An initial audit of the record of EbE involvement in teaching was conducted by requesting information by email from 13 course teaching leads regarding the number of teaching sessions that included EbEs, and details of the format of involvement.

The online survey platform, Qualtrics, was used to create and store answers from two separate survey questionnaires that were provided to the PEG and trainees respectively. Each survey began with an initial participant information sheet and consent form that participants were required to complete before progressing to the full survey.

The PEG survey included a demographics questionnaire which was followed by 15 questions that were underpinned by the Ladder of Participation.^21^ Therefore, questions targeted the broad themes of ‘level of involvement’ (e.g., ‘what kind of way were you involved in teaching?’), ‘impact’ (e.g., ‘How might PEG involvement in teaching impact PEG members?’) and ‘improvement’ (e.g., ‘how would you like PEG involvement in teaching to change?’) (Appendix B).

The trainee survey included 10 questions that were underpinned by Kolb's Learning Cycle.^17^ In this way, the questions targeted the different stages of Kolb's reflective learning cycle, for example, ‘Please explain how EbE have been involved in teaching’ (concrete experience), ‘How have you found the experience?’ (reflective observation), ‘What have you learnt about your own practice from EbEs in teaching?’ (abstract conceptualisation), and ‘How has EbE involvement changed your attitude, behaviour and practice?’ (active experimentation) (Appendix C).

### 2.4 Data analysis

The thematic analysis procedure described by Braun and Clarke^22^ was used to manually code and analyse key themes in survey responses. The PEG and trainee surveys were analysed separately.

The free text for each survey was read and reread to ensure familiarisation. Key words or phrases were highlighted, and the ‘comment’ function in Word was used to develop initial codes alongside the text data. These codes were then organised into categories of repeated patterns, or themes. An inductive approach to analysis was taken, to ensure that the themes would closely reflect participants’ experiences rather than be driven by specific research questions. Provisional themes were reviewed to check that they (a) reflected the codes, and (b) reflected the semantic content of the data set as a whole. Interpretations and conclusions were reviewed and discussed in meetings with the research team. The research team was made up of a trainee, PEG member and course tutor. It was therefore important for them each to reflect on their own positions in relation to the topic and how this might influence their interpretation of the data. Where possible, the words used by participants were included in the theme title. Quantitative data derived from the two surveys were summarised by descriptive statistics.

## 3 RESULTS

The initial audit of EbE involvement in teaching revealed that 9 out of the 13 teaching streams involved EbEs in teaching in some way. The teaching areas covered were: ‘Equality, Diversity and Inclusion’, ‘Working Age Adult’, ‘Research’, ‘Older Adult’, ‘Neuropsychology’, ‘Health’, ‘Forensic’, ‘Intellectual Disability’ and ‘Advanced Therapeutic Interventions’. Types of involvement included: (1) EbEs talking about their experiences of certain conditions, and the processes of receiving diagnoses and psychology involvement, (2) EbEs co‐designing and co‐facilitating the teaching sessions and (3) EbEs facilitating small group discussions. Two of the 13 teaching leads contacted were not able to provide any data due to recently being appointed to the post and therefore not having access to previous records of EbE involvement.

Overall, the audit demonstrated that there was no current centralised or agreed upon method for recording data about EbE involvement, and the level and detail of data recorded varied across teaching.

### 3.1 PEG survey

Of the 14 PEG members who were provided information about the study, 12 (83%) consented to complete the survey. Two of these 12 did not continue past the initial consent stage, leaving 10 participants who completed the survey in full. The majority were female (60%), aged 36+ (90%) and identified as white (80%). Seven had been PEG members for 1–2 years and three for 10 years. Eight considered themselves to have a mental health difficulty, seven a physical health difficulty and three stated they were caring for someone with a mental health difficulty. Five had completed a bachelor's degree and one a doctorate degree. Four were retired, three were working part‐time, one was self‐employed and one was a student. The majority identified with a religion (six Christianity, two Islam) and five of these were actively practising.

The initial questions on the survey asked about their level of involvement in teaching in the 2020 academic year. Seven PEG members stated that they had been directly involved in teaching, with the majority having heard about involvement through a course email or PEG meeting. They identified types of involvement as ‘talking about lived experience in large or small groups’, ‘co‐presenting’ and ‘leading small group discussions’. Three PEG members stated that they had not been made aware of the opportunities available for involvement.

Thematic analysis yielded four themes: (1) ‘informing change’, (2) ‘bringing purpose to experiences’, (3) ‘educating by ‘making it real’ and (4) ‘ensuring empowerment’. The fourth theme encapsulated three subthemes: ‘flexible opportunities’, ‘keeping EbEs informed’ and ‘potential for distress or discomfort’. These themes are presented below and supported by excerpts from the survey.

#### 3.1.1 Informing change

Eight (80%) of the PEG members surveyed, commented on a motivation to bring about change in themselves, the trainees, and future clients. Two EbEs also named specific clinical areas in which they hoped to influence through involvement.

I hoped that, by discussing good and bad experiences, we could influence future outcomes for clients in a similar position to us. (EbE 3)

#### 3.1.2 Bringing purpose to experiences

All 10 PEG members wrote about the process of making sense of their lived experiences through participation in teaching, and how this creates purpose and builds their self‐confidence.

This teaching helps me both to appreciate the journey I have been on, the extent of my knowledge, gives me confidence… (EbE 1)

Four PEG members wrote about the role of involvement in developing their own interests and knowledge.

… I personally have benefited from being involved…helping my own research. (EbE 6)

#### 3.1.3 Educating by ‘making it real’

Seven PEG members (70%) felt that involvement helped trainees reflect on lived experiences, to a greater extent than in other more theory‐based lectures.

Lived experience says more than just reading from a book, people can relate. (EbE 10)

A couple of PEG members also commented on the value of the interactions between trainees and EbEs in lectures.

Clinical psychologist trainees can learn a lot from people with experience and ask questions. (EbE 8)

#### 3.1.4 Ensuring empowerment

All 10 PEG members commented on the importance of considering steps to ensure EbEs feel empowered in teaching. Within this overarching theme, three subthemes highlighted factors that PEG members felt influenced this sense of empowering.

### 3.2 Flexible opportunities

Several PEG members felt that further training in teaching delivery, including training in using ‘Zoom’, would support them and their future careers.

Getting more training and certificates that may help us getting into employment or education in the future. (EbE 10)

The ability to join teaching sessions online was also thought to be an important factor for increasing access, for one PEG member:

Virtual access has enabled me to be involved, without concern about accessibility issues and travel. (EbE 6)

### 3.3 Keeping EbEs informed

Three PEG members commented on the importance of having information about the teaching session beforehand and a space to debrief afterwards.

It is helpful to always have a pre‐meeting to meet the tutor and discuss and plan how you are going to be involved. (EbE 4)

### 3.4 Potential for distress or discomfort

All PEG members felt there was potential for discomfort or distress for the EbEs involved in teaching, due to the nature of the content or the questions asked.

…although there may be slightly uncomfortable moments when boundaries are challenged and pushed. However, that generally means one is learning. (EbE 1)

One PEG member felt that the responses of trainees and co‐facilitators during teaching had an impact on their experience.

If the students and tutors seemed uninterested, there was no positive feed‐back from them. (EbE 2)

### 3.5 Trainee survey

Of the 30 trainees who were provided with information about the study, 27 (90%) consented to complete the survey, and 20 of these continued with the survey after consenting. Nineteen completed the survey in full, and one trainee partially completed the survey.

Thematic analysis yielded five themes: (1) ‘Connecting with “lived realities”’, (2) ‘How to be a better clinical psychologist’, (3) ‘Involvement consolidates learning’, (4) ‘Emotional impact’ and (5) ‘Integrating EbEs into teaching’. Each theme encapsulated several subthemes which are described below and supported by excerpts from the survey.

#### 3.5.1 Connecting with ‘lived realities’

Most of the trainees (75%) commented on their gained insight and understanding of experiences from hearing about the ‘lived realities’ of EbEs in teaching. They emphasised the value of connecting on an emotional level with EBEs, in a way that is less possible in other teaching. They also reflected on how this influenced them and built their confidence as professionals.

Hearing from a carer & a person with learning disabilities was a really important moment in teaching for me, as this is an area that I don't know well and felt intimidated by. Hearing from EBE normalised and humanised what had up until that point had felt theoretical and distant, and made me more confident going forward. (T7)

### 3.6 The whole journey

A number of trainees emphasised the value of hearing about the ‘journey’ of the EbE, in terms of the onset and development of difficulties, rather than just focusing on the here and now.

It has made me more appreciative of people's journeys before they come to see psychology, for example have they got a child with a learning disability, did they go through a mental health crisis in a different country, have they had a life changing injury? (T21)

### 3.7 Permission to be curious

Trainees valued the opportunity to speak more informally with EbEs in small groups and have permission to ask questions.

They are so powerful and insightful and it's such a privilege to hear people's narratives and be able to ask questions… They're often the things that would be difficult to ask someone in session so the chance to have this is really valuable. (T14)

#### 3.7.1 How to be a better clinical psychologist

Fifteen Trainees (75%) identified a range of ways in which EbE involvement in teaching supported them to develop their skills as clinical psychologists.

### 3.8 Putting learning into practice

They wrote about the importance of EbEs in giving them knowledge that can be translated into the clinical setting.

I also found it helpful to hear some of their more negative experiences of services, as I have tried to bear those in mind and avoid similar practice on placement. (T3)

### 3.9 Specific and nonspecific skills

Trainees felt that EbEs helped them build an understanding of developing comprehensive assessments and formulations of clients.

It has highlighted some of my blindspots in assessments (what I don't think to ask), enhanced my formulation skills… particularly in terms of relationship to help for people who have had negative experiences of services. (T18)

At the same time, a number of trainees emphasised their learning around the importance of nonspecific skills such as empathy and listening, which they felt was less present in other lectures.

It reminds me to be a human. With all the complex formulation and evidence bases we learn, it is sometimes easy to forget the softer skills of compassion, empathy and kindness, which are arguably more important than the theory… (T9)

### 3.10 Systems

Trainees commented on their growing understanding of the importance of working with systems around clients, as a result of the EbE involvement in teaching. Five trainees felt they had gained insight into the role of carers and the importance of supporting them as well as the clients.

Hearing from carers as well as the EbE themselves has encouraged me to think about how to support not just individuals, but whole families which may be impacted. (T2)

A greater understanding of organisational issues was also named by trainees who felt they had learned more about how these systems can be a barrier to care.

… I have learned more about the difficulties and barriers that people face with regards to our national health service, which is depressing at times. (T4)

#### 3.10.1 Involvement consolidates learning

Forty percent of trainees wrote about the impact that EbE involvement had on the quality of their learning and memory for the information discussed.

### 3.11 Emotional learning

Eight trainees talked about the role of emotional learning in promoting their ability to remember and act on teaching that had involved EbEs.

It is often an emotional interaction to hear from EbEs. It has been difficult to listen to some of them, but they are the teaching sessions that stand out in my mind as being the most powerful and we have the most to learn from. (T21)

### 3.12 Reflecting on assumptions

Three trainees shared that hearing from EbEs had helped them reflect on their own assumptions about different client groups, influencing their approach in clinical settings.

Having one EbE discussing their experience of dementia hugely challenged my preconceptions of individuals living with dementia…I really tried to bear this learning in mind when delivering post‐diagnostic groups for people with a recent diagnosis of dementia, and felt it enabled me to instill hope with more conviction… (T3)

#### 3.12.1 Emotional impact

Sixty‐five percent of trainees recognised the impact of the emotional content of lectures involving EbEs on themselves, both personally and professionally.

### 3.13 Reigniting passion and striving for change

Nine trainees spoke about having their passion for the clinical psychology role reignited after hearing from EbEs. They linked this to a desire to bring about positive change as professionals, and to the systems they work in.

It has reminded my why we do the job that we do. I find after sessions with EbEs I have a real fire in my belly again! (T21)

*…*often the stories told by EbEs makes me feel sad about the state of our health service, the barriers and obstacles faced by our clients. These are however important issues to highlight, and as young trainees it facilitates an attitude of striving and hoping for implementing change within these systems. (T4)

### 3.14 Support for trainees

Six trainees wrote about finding some EbE teaching sessions emotionally challenging to ‘sit with’. One trainee suggested a need to support trainees following these sessions, and another felt it was helpful when lecturers prepared them for emotional content and gave them time to process it following lectures.

*…*I think sometimes stories can be harrowing, and it's really useful for the session leader to name this, and make sure we take time out for ourselves after meeting them (i.e. by putting the stories just before lunch, etc). (T9)

While being exposed to these feelings of distress was felt to be crucial by one trainee, they also recognised the limits of online teaching in ensuring trainees connect with these experiences.

… it can be hard to sit with that emotion on Zoom vs in the room, although I think it is important for us as trainees to be exposed to these discussions early in training. (T15)

#### 3.14.1 Integrating EbEs into teaching

There was an overarching theme of trainees (70%) considering the process in which EbEs were involved in teaching sessions.

### 3.15 Disparity of involvement

Ten trainees made reference to a disparity in level and type of involvement across streams and client groups, stating hopes of hearing more wide‐ranging perspectives.

Would be nice to hear from some children and young people… (T4)

…it would have been really helpful to hear from individuals with lived experience who may not quite fit v specific diagnostic criteria, or who may have had alternative reflections on diagnoses. (T3)

### 3.16 Tokenism and power

Trainees reflected on the power balance between EbEs and the other facilitators in teaching. While some trainees noted strengths in co‐delivery, others felt more could be done to ensure the roles were shared more evenly between facilitators.

The lecturers always placed a great emphasis on them being co‐deliverers with an even power dynamic. (T7)

…wondered why they couldn't be involved in delivering material, rather than only being able to speak about their direct experiences?… Sometimes I felt like they were being exhibited, for us to ask nosy questions. (T13)

### 3.17 Ensuring time for reflection

An important aspect of integrating EbEs into teaching, noted by five trainees, was the need to ensure trainees had time to reflect on and process what they had heard.

*…* it would be useful if more tasks ask us to take account of what we have learned from an EbE/ask us to reflect on what we might do differently after listening to an EbE share their experiences. (T18)

### 3.18 Support for EbEs

Eight trainees considered ways of supporting EbEs to ensure they had a helpful experience of teaching. They recognised the possibility of EbEs feeling uncomfortable when asked certain questions, and therefore the importance of trainees and EbEs agreeing on how to navigate challenging questions.

…it was always helpful to navigate this discussion with some ‘ground rules’ (e.g. saying that they can simply choose not to answer any questions that feel too personal/uncomfortable). (T15)

One trainee felt that EbEs needed further support with online tech support during teaching sessions.

… a little more time needed to be given to supporting EBEs with the tech required for online teaching…it would have been useful for session leaders to have a practice run with them to make sure the tech is working smoothly, as sometimes we got less time with the EBEs due to tech issues. (T9)

## 4 DISCUSSION

This study aimed to examine the level and impact of EbE involvement on a UK DClinPsych course to ensure a meaningful process for those involved. The findings below are discussed in relation to the three research questions; (1) ‘How are EbEs currently involved in teaching on a DClinPsych course?’, (2) ‘How are EbEs and trainees impacted by EbE involvement in teaching?’ and (3) ‘What do EbEs and trainees think could be improved with regard to EbE involvement in teaching?’

The initial audit revealed that records of involvement varied in quality across teaching, with some gaps in evidence regarding whether EbEs were involved at all in some teaching areas. The lack of consistent record keeping meant that it was not possible to report on the number of lectures that involved EbEs, however, nine of the 13 teaching streams were found to involve EbEs in some way. Levels of involvement varied, with some EbEs choosing to talk about their personal experiences, and others co‐presenting teaching material or being more actively involved in the design and production of a lecture. At the same time, some EbEs reported to have not been made aware of their options for involvement. Therefore, the degree to which power was shared, and therefore the level of participation,^8^ appeared to vary across teaching and EbE.

The PEG survey showed that involvement such as sharing personal experiences or answering trainee questions promoted self‐confidence and a sense of purpose from creating positive change for others. The PEG survey also highlighted the need for EbEs to be fully informed and supported in teaching, through the provision of information, debrief meetings and further training where appropriate. Therefore, while these findings are consistent with other studies showing that service user involvement in mental health systems has therapeutic benefits for EbEs,^23^ there is also a need to ensure they are properly supported to ensure their involvement lands higher on the ladder of participation.^8^ These results align with similar studies of EbE involvement in health education^24^, ^25^ which emphasise the importance of sensitively involving EbEs in teaching processes to ensure the processes are purposeful and not tokenistic.

The trainee survey indicated that EbE involvement in teaching promoted a Kolb reflective learning process (1984) by firstly exposing trainees to personal experiences which they then reflected on, through discussion and personal reflection, to guide their future practice. The trainees felt they gained insights into the skills and knowledge required for practicing as clinical psychologists, including assessment and formulation skills, systemic working and nonspecific skills such as empathy, normalising and validation. Additionally, trainees reflected on having actively made changes to their clinical approach (Active Experimentation)^17^; following teaching with EbEs. In line with research,^18^ the trainees felt they remembered more from teaching involving EbEs due to having a greater emotional connection with the material covered. Similar to the PEG responses, trainees felt that EbE involvement could be distressing and that both EbEs and trainees should be better supported by creating clear ‘ground rules’ for EbE sessions, and incorporating more time for reflection. In line with the audit, trainees felt there was some disparity in level of involvement from different patient groups across teaching, and felt this was an area for improvement, to move closer to co‐production.

### 4.1 Limitations

The survey methodology, while chosen purposely to allow access to a greater number of participants, was limited with regard to the level of exploration afforded around each question. Additionally, recruitment of EbEs through the PEG is likely to have reduced the heterogeneity of the sample, as many of them were highly educated and interested in research. There is therefore scope for future research to incorporate wider recruitment strategies and utilise interview methods to enhance the current findings.

The analysis and interpretation of the results are likely to have been influenced by the positions and perspectives of the researchers. The team worked closely to ensure that the codes and themes closely matched the extracts to ensure that important aspects of the data were not overlooked.^26^ Finally, the attrition from consent to completion may have been the result of the participants forgetting to finish the survey once started. This therefore could have been minimised had the researcher provided further prompts to encourage completion.

### 4.2 Clinical implications and future directions

The lack of recorded data with regard to EbE involvement in teaching, suggests a centralised and agreed up system be created for inputting data related EbE involvement. This should include both what is offered and what is taken up. This would ensure that EbEs are afforded choice over the position and role they wish to hold.

Based on the survey feedback, it is recommended that the service consider ways of increasing the variety of EbE perspectives included in teaching, to encourage richer reflections and learning. Steps to promote the wellbeing of EbEs and trainees should include planning and debriefing meetings for EbEs and greater time for reflection for trainees. It is also recommended that trainees be made aware of EbE involvement and appropriate areas for discussion in advance. It may be appropriate to consult the PEG group about opportunities for training, such as in technical support if necessary.

## 5 CONCLUSION

PEG members and trainees showed considerable agreement with regard to the impact of EbE involvement in teaching. Issues around methodology and sampling have guided suggestions for future research. Recommendations for the service are hoped to enhance the training of clinical psychologists and therefore benefit future clients and services they work with.

## CONFLICT OF INTEREST STATEMENT

The authors declare no conflict of interest.

## ETHICS STATEMENT

According to the HRA decision tool, this project falls into the category of service evaluation/audit. Therefore, NHS approval was not required. Service evaluation approval will be sought before commencing the project.

## Data Availability

The data that support the findings of this study are available from the corresponding author upon reasonable request.

## Appendix A PEG 3‐YEAR PLAN

**The People's Experience Group 3 Year phase Plan**

**Table:**

Year 1**—**2020/2021	Year 2**—**2021/2022	Year 3**—**2022/2023
Design and embedding	Improving	Maintaining and continue move towards co‐production
The PEG aims to develop a selection process for new membership, with the aim of increasing diversity and experience within the group. The course will run an open day for interested parties to hear more about the role. An information sheet and role specification will be developed and shared with interested parties. Following this, a one‐to‐one meeting will be held and if it's felt by both parties that joining the PEG would be of benefit, a 3‐year term of office will begin. This includes an induction training session and induction pack. New members will be allocated a mentor (who will initially be a course staff member but as the PEG is further established, experienced PEG members will be mentors). The PEG will meet quarterly and will comprise: – Approximately 8 people with personal experiences – 2 course staff – one trainee from each year (minimum) There will be PEG representation in all streams. Academic (co‐facilitating teaching), clinical (attendance at the practice and placement committee) and research (attendance at PAS, consulting on trainee research projects and attendance at the research committee). A PEG member will be represented on each course committee (including admissions) and will feedback to the PEG group quarterly. A PEG member will also attend GTiCP.	The second year of the PEG implementation will focus on building on the initial progress from year one. This includes: *Research* PEG members with an interest and/or experience in research will act as collaborators/co‐supervisors (as appropriate) for trainee research projects. This will include training for PEG members interested. The PEG will have greater involvement with trainee research through informal lunches following PEG meetings. Trainees can join the lunch to discuss their project and research ideas in a less formal setting with members, to share ideas and promote collaboration. *Academic* A PEG member will be linked in with each stream lead to provide input when reviewing the content of teaching for the following year, and to review trainee's feedback on teaching for each stream. *Admissions* The aim by this stage is to include a PEG member in the video task design and production. In addition, we plan to have a PEG member available each day at interviews to welcome applicants and to be involved in invigilating the video task.	Further aims for the final phase include an increase in PEG presence for admissions, with a PEG member on each panel for clinical interviews and a member working with the video task team. PEG members will also sit on interview panels for new course staff. In addition, the formation of a young person's PEG will begin. The aim is to establish a small (3–4 person) group who meet biannually comprising young people and young carers. The remit of this group will be much smaller than the original PEG. By this phase the aim is to recognise and measure the impact of the existing PEG group through a PEG annual report and research project. PEG members who were recruited in 2020 will be reaching the end of their term of office and future recruitment and signposting will be considered. Finally, future aims will include a PEG conference and publication of any research projects. PEG members will be paid for their time in accordance with Oxford Health FT guidelines.

## Appendix B PEG SURVEY QUESTIONS

**Table:**

*Identifying levels of participation*
Were you made aware of opportunities available to you for getting involved with teaching on the DClinPsych course in 2020–2021?
If applicable, how did you hear about the types of opportunities available? (please state ‘n/a’ if not applicable)
Were you involved in teaching in the 2020‐2021 academic year?
If yes to q 3: In what capacity were you involved?
Please can you expand on your answer (we want to understand more about your individual experience)
What made you decide to get involved?
If you were not involved, please state your reason for this
Is there anything else that you feel would be important to share with us about any reasons for or barriers to getting involved with teaching?
*Impact*
Do you think it is important for PEG members to be involved in some way with teaching prospective clinical psychologists?
How do you think PEG members can help improve the teaching experience for prospective clinical psychologists?
What do you think is the positive impact for PEG members specifically, for being involved in teaching?
What do you think is the negative impact for PEG members specifically, for being involved in teaching?
*Improvement*
Would you like to be involved in teaching in a different kind of way than is available to you now?
If applicable, please explain how would you like PEG involvement in teaching to change and what difference this would make to you?
What else might improve the way PEG members are involved in teaching? Please explain your answer

## Appendix C TRAINEE SURVEY QUESTIONS

**Table:**

*Concrete experience*
How were experts by experience involved in your teaching?
*Reflective observation*
How have you found the experience of experts by experience being involved in teaching?
Overall, how satisfied were you with the involvement of experts by experience in teaching?
Overall, how much would you say you have benefitted from experts by experience being involved in teaching?
What worked with regard to experts by experience being involved in teaching?
What didn't work with regard to experts by experience being involved in teaching?
How are you impacted emotionally by expert by experience involvement in teaching?
*Abstract conceptualisation*
What have you learnt about your own practice from expert by experience involvement?
What could be improved with regard to EbE involvement in teaching?
What would be the impact of these changes, for you as a trainee?
*Active experimentation*
How has EbE involvement in teaching changed your attitude, behaviour or practice?
